# A Comprehensive Review of Pregnancy in Sickle Cell Disease

**DOI:** 10.7759/cureus.41165

**Published:** 2023-06-30

**Authors:** Tejas Shegekar, Sandhya Pajai

**Affiliations:** 1 Obstetrics and Gynecology, Jawaharlal Nehru Medical College, Datta Meghe Institute of Medical Sciences, Wardha, IND

**Keywords:** vaso-occlusive crisis, feto-maternal outcome, sickle cell crisis, sickle cell disease (scd), adverse pregnancy outcome

## Abstract

Sickle cell hemoglobinopathies encompass a range of qualitative and quantitative hemoglobin disorders that are inherited genetically. This group of disorders includes sickle cell beta thalassemia, sickle cell trait, and sickle cell disease (SCD). Globally, SCD is the most common disorder. Even epidemiological data suggests the majority of diseases, as well as traits, are concentrated in Sub-Saharan Africa, North-East Africa, the Middle East, and India. The physiological changes in pregnancy predispose to an increased risk of catastrophic events like a vaso-occlusive crisis, thromboembolic events, and their related sequelae, leading eventually to villous infarction, necrosis, and fibrosis leading to compromising uteroplacental circulation. Conversely, the mother may exhibit exacerbated symptoms of gestational hypertension, placental abruption, preterm labor, and venous thromboembolism. Although this disease is manageable, it has the potential to adversely impact maternal and child health on a national level. The chances of severe complications in the pregnant state affecting both mother and fetus attract due attention of health services towards redefining and researching this disease and its management frequently. The literature review on the following situation advocates the general treatment to be observed under the headings of preconceptual care, strengthened antenatal care, strict intranatal care, and compliant post-natal care. Preconceptually, genetic screening of couples, with education on the adverse effects of the disease, comes as the first line of management. Newer facilities like preimplantation genetic diagnosis and celocentesis may even allow for early diagnosis as well as help patients who do not wish to terminate the pregnancy by selective transfer of unaffected embryos. This may be combined with an extensive evaluation of the psychosocial aspect and socioeconomic status of couples who administer vaccines as prophylaxis for preventable diseases. Strengthening antenatal care is associated with routine blood investigations for every registered antenatal patient with adequate awareness about the conditions that precipitate the crisis. All patients should be prophylactically treated with appropriate doses of aspirin, iron, folic acid, and multivitamins. Radiological examinations by ultrasonography may be used to monitor placenta previa, abruption, or preterm labor. Later in pregnancy, it should be recommended to perform biophysical profiling and assessment of umbilical artery flow. Intranatal care deals with strict-term institutional delivery of all sickle cell-diseased mothers with a preference for vaginal delivery. Post-natal care requires a precise assessment of blood loss during labor to initiate transfusion therapy as soon as needed. Exclusive breastfeeding, with the importance of early initiation of it, must be emphasized. Screening of neonates as quickly as possible must be done for hemoglobinopathies. Through this review, authors are trying to make aware of the complications that can be faced during pregnancy in SCD patients, its prevention, and its treatment according to various new guidelines and research available.

## Introduction and background

Sickle cell hemoglobinopathies are a group of genetically inherited disorders caused due to point mutation in the β-globin gene present on chromosome eleven, which result in the substitution of valine in place of glutamic acid at the sixth position of the β-chain of normal hemoglobin [1]. Sickle cell hemoglobinopathies is an umbrella term that includes three main conditions: sickle cell-β-thalassemia, sickle cell trait, and sickle cell disease (SCD) [2]. Sickle cell-β-thalassemia is an inherited form of SCD that affects red blood cells both in the production of abnormal hemoglobin, as well as the decreased synthesis of β-globin chains [3]. Sickle cell trait is a heterozygous condition with a sickle cell mutation in one β-globin chain gene and another is normal. In contrast, SCD is a homozygous condition in which there is a sickle cell gene mutation in both the β-globin chain gene resulting in sickle cell anemia [4].

**Figure 1:**
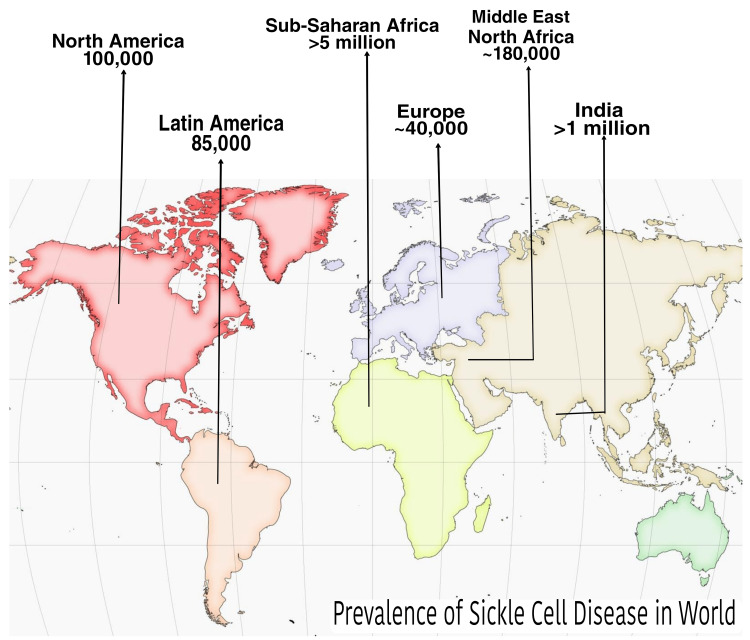
Prevalence of sickle cell disease in the world [5] Figure created by authors

SCD is one of the most common inherited genetic and life-threatening disorders affecting more than 6.4 million people worldwide [5]. Pregnancy exacerbates SCD resulting in severe complications for the mother and fetus and also increases the chances of maternal and fetal death [6]. There are approximately 6.4 million people living with SCD worldwide (Figure 1), among which 0.1 million are in North America, 85,000 in Latin America, more than 5 million in Sub-Saharan Africa, approximately 0.18 million in the Middle East, and more than 1 million in India [7,8]. About 300 million people have sickle cell trait, and approximately 0.3 million children are born yearly with SCD worldwide. In the second to fifth decade, the 21st-century incidence of children born with SCD is expected to increase by 30% worldwide [8]. Sub-Saharan and North-East Africa, India, and the Middle East are the main hubs of SCD in the world. Sub-Saharan Africa has the highest prevalence as well as complications of SCD in pregnancy, with a maternal mortality rate of 0.38 to 1.29 per 100,000 births and a perinatal mortality rate of 1.21 to 2.50 per 100,000 births [9].

In the United States, 1 out of every 365 Black or African American-born children and 1 out of every 16,300 Hispanic American-born children have SCD [7]. The prevalence of SCD in children less than one-year-old was found to be 500-2000 per 100,000 births in Sub-Saharan African countries, 20-500 per 100,000 births in South America, and less than 500 per 100,000 births in the USA and Europe [10].

India is home to the world's largest population of tribal communities. According to data collected by the Ministry of Home Affairs, Government of India, during the 2011 census, 8.6% of the total population of India, which is about 67.8 million, is tribal. Among the different tribal groups, the prevalence of sickle cell carriers varies between 1% and 40% [11,12]. This review aims to provide a comprehensive understanding of the pathophysiology of SCD, early identification of complications, enhancement of management strategies as well as guidance for further research.

## Review

Methodology

**Figure 2:**
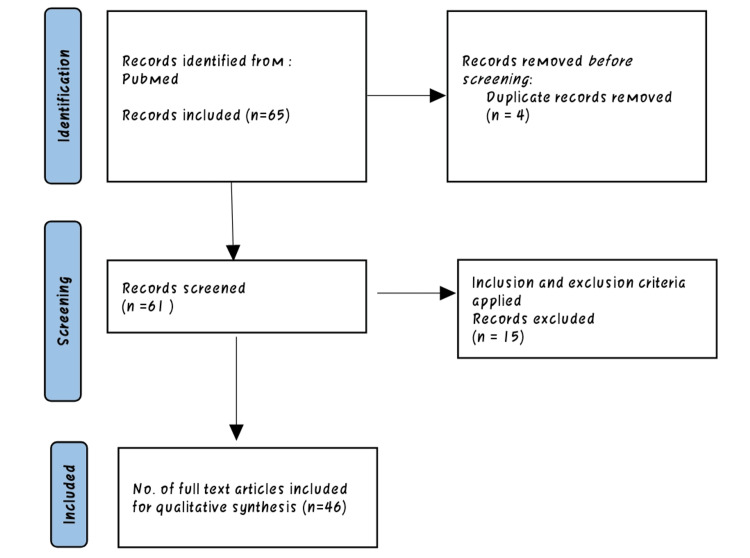
A flowchart of the methodology used for the review

The search methodology followed a systematic approach to identify relevant studies for the review. The process involved searching various databases, defining inclusion and exclusion criteria, screening articles, and selecting the final studies for the review. A comprehensive search was conducted in electronic databases, including PubMed. These databases were chosen to ensure broad coverage of relevant literature. The search included studies published from the inception of the databases up to the present day, with no specific date restrictions. This ensured the inclusion of the most up-to-date research on the topic. The search strategy incorporated a combination of key terms and Medical Subject Heading (MeSH) terms related to SCD, pregnancy, pathophysiology, and associated complications. The key terms used included "sickle cell disease," "pathophysiology," "pregnancy," "complications," and relevant synonyms. Studies were included if they investigated the pathophysiology of SCD during pregnancy, identified complications, or provided insights into the management of the condition. Only articles published in English were considered. Case reports and editorials were excluded from the review. The initial screening involved reviewing the titles and abstracts of the identified articles based on the inclusion and exclusion criteria. Full-text articles were obtained for the potentially relevant studies, and further screening was conducted to select the final articles for the review. A total of 46 articles met the inclusion criteria and were included in the final review. The following Preferred Reporting Items for Systematic Reviews and Meta-Analyses (PRISMA) flow diagram (Figure 2) provides a visual representation of the search methodology, showing the number of articles identified, screened, and included in the final review.

Pathophysiology of sickle cell disease

SCD results from the point mutation in which hydrophobic valine is substituted in the place of hydrophilic glutamic acid at the sixth position of the β-chain of normal hemoglobin. When there is decreased state of oxygen concentration, these irregular structures that have yielded due to mutation interacts with other hemoglobin S (HbS) molecules resulting in the formation of polymers [13]. These HbS polymers rapidly grow into long filaments that increase cell stiffness and deform the red blood cell (RBC) membrane resulting in characteristic “sickle-shaped” RBCs. Repeated injury to RBC results in the hemolysis of RBCs and resultant sickle cell anemia, cellular energy depletion, and stress [13,14]. Normal RBCs travel smoothly through small blood vessels, but sickle cell RBCs do not have this capability and result in occlusion of blood flow and reduced blood flow to body tissue distal to the blockage. This vascular occlusion results in a painful crisis [13,15]. Other factors that contribute to SCD vascular disease include an increase in the level of proinflammatory mediators like interleukin-1β, interleukin-8, and tumor necrosis factor-α; increased adhesion of delicate sickle cells to endothelial cells, circulating white blood cells, and blood platelets [16].

Effect of pregnancy on SCD pathophysiology

The physiological changes which take place in pregnancy, such as an increase in metabolism, blood stasis, and coagulability, aggravate SCD leading to an increased risk of adverse effects for SCD mothers and neonates [17]. This increased risk can alleviate complications such as vaso-occlusive crisis, acute chest syndrome (ACS), bone necrosis, and thromboembolic events. Any underlying cardiopulmonary disease may manifest when the woman is exposed to the physiological stress of pregnancy. In SCD, acidic conditions decrease the oxygen affinity of abnormal HbS, promoting its polymerization. This leads to the formation of long fibers, distorting the shape of RBCs and causing them to assume the characteristic sickle shape. These sickled cells can obstruct blood vessels, impair blood flow, and contribute to the complications associated with the disease. Vaso-occlusive events in the placenta may trigger infarction, villous necrosis, and fibrosis, thereby reducing uteroplacental circulation, resulting in chronic fetal hypoxia and adverse fetal outcomes [17-20].

Effect of SCD on maternal and fetal health

Sickle cell anemia is one of the most familiar inherited diseases in the hemolytic trait family. It attacks the interior structure of RBCs, affecting how well they carry oxygen across the body. The process responsible for instructing the body to synthesize the iron-rich protein called hemoglobin, which is predominantly found in RBCs, is altered in sickle cell anemia. RBCs are capable of oxygen transport from the lungs to various tissues due to the presence of hemoglobin. Hence sickle cell anemia causes various complications during pregnancy as the condition is related to changes in hematological constituents like hemoglobin levels, RBC indices, and reticulocyte count. Hypertension and thrombosis due to endothelial dysfunction/damage, and inflammation during pregnancy are made more likely by sickle cell anemia. Infertility, early birth, and having children with low birth weights are all risks that can be increased by it. Maternity may make SCD worse and increase the frequency of symptoms. Skeletal systems and joints are where pain attacks typically occur. Some endure for weeks, although they can last anywhere from a few hours to several days. At the time of pregnancy, sickle cell disease may raise your danger of miscarriage and preterm birth (less than 5 pounds, 8 ounces) [21]. Infertility may increase the risk of obstetric complications for women, particularly others with sickle cell anemia since it has been linked to the worsening of SCD. Elevated cardiac output in order to lower systemic arterial resistance is one of the physiological changes that occur during pregnancy. Progesterone-related changes in stroke volume, respiratory minute volume, inspiratory reserve, inspiratory reserve, and total pulmonary resistance may endanger oxygen supply. Additionally, hypokalemia emerges, enhancing the fetal exchange of gases but perhaps affecting RBC hemolysis. Dilutional anemia is linked to increased plasma levels during pregnancy; however, there are no data demonstrating how much baseline anemia is aggravated in women with sickle cell disorder who might have persistently expanded plasma prior to pregnancy [19].

Women with sickle cell illness do not appear to experience an increased frequency of antenatal and postnatal bleeding. Chronic anemia and maternal complication caused by ischemic injury are two factors in SCD-affected women that may have an impact on embryogenesis. When comparing specific studies, maternal and fetal adverse outcomes are significantly lower in developed high-income nations than in low-income ones [22]. Still, when comparing odds ratios for aggregated bad results, both high as well as low-income countries have similarly high odds ratios [23]. Obstetrical problems range widely, although hypertensive disorders, poor fetal growth, premature labor, and maternal or perinatal mortality seem to be regularly reported throughout both retrospective and longitudinal epidemiological studies such as cross-sectional medical management databases. Even though the specific etiology is uncertain, hypertensive problems of pregnancy, which are common in women with SCD, may be caused by endothelial dysfunction, swelling, and perinatal infarction [24]. The chance of miscarriage is arguably the most concerning. A 500-person cohort study did not discover an elevated risk for neonatal mortality in sickle cell trait (SCT) patients [25]. Maternal and fetal outcome and period-wise hematological complication in SCD mother is summarized in Table 1 and Table 2 respectively [19,24,26].

**Table 1:** Maternal and fetal outcome in sickle cell diseased patient SCD: sickle cell disease [19,24,26] Table created by authors

Maternal complications in SCD	Fetal outcome
Increased maternal death	Perinatal mortality
Increase in the hypertensive disorder of pregnancy- pre-eclampsia and eclampsia	Intrauterine growth retardation
Miscarriage	Low birth weight
Placental abruption	Preterm delivery
Placenta previa	Prematurity
Toxaemia	
Preterm labor	
Retained placenta	
Postpartum hemorrhage	
Bacteriuria	
Painful crisis	
Acute chest syndrome	
Urinary tract infection	
Sepsis	
Venous thromboembolism	
Acute exacerbation of anemia	
Proteinuria, worsening of renal disease	

**Table 2:** Period-wise hematological complication HELLP: hemolysis, elevated liver enzymes, and low platelet count Table created by authors

Period	Complications
First trimester	Vaso-occlusive crisis, hemolytic anemia
Second trimester	Vaso-occlusive crisis, transfusion reaction, hemolytic anemia
Third trimester	Vaso-occlusive crisis, pre-eclampsia, eclampsia, HELLP syndrome, transfusion reaction, hemolytic anemia
Perinatal period	Thrombotic event, HELLP syndrome, disseminated intravascular coagulations, postpartum hemorrhagia

Treatment

Preconceptual Care

General evaluation: All females and males with SCD at childbearing age should be aware of various risks and adverse effects of SCD on pregnancy to optimize general health and decrease reproductive risks [27]. Screening parents and genetic counseling is the main aim of preconceptual care [28]. Antenatal screening of patients with SCD by 13 weeks of gestation allows couples to decide whether they want to continue or terminate pregnancy [28,29,30]. Celocentesis at 7 to 9 weeks of gestation also allow for earlier diagnosis [25]. Preimplantation genetic diagnosis (PGD) is an alternative option for couples who do not want to terminate a pregnancy. It involves the selective transfer of unaffected embryos after in-vitro fertilization (IVF) [31]. Females presenting with any signs or symptoms of the respiratory or cardiac system must be evaluated thoroughly, and further reproductive plans should be developed accordingly. Patients using drugs like ACE (angiotensin-converting enzyme) inhibitors and ARBs (angiotensin II receptor blockers) must be advised to discontinue before conception, as well as hydroxyurea must be advised to discontinue 6 months before pregnancy (Table 3) [27,32,33].

**Table 3:** Potential teratogenic drugs use in the treatment of sickle cell disease ACE: angiotensin-converting enzyme ARB: angiotensin II receptor blocker Table created by authors

Drugs	Recommendations
Hydroxyurea	Stop six months before conception or immediately on pregnancy confirmation
ACE inhibitors/ARBs	Stop before conception
Injectable iron chelators	Avoid during pregnancy
Oral iron chelators	Avoid during pregnancy
Voxelotor	Stop before conception or immediately on pregnancy confirmation
Crizanlizumab	Stop before conception or immediately on pregnancy confirmation
Glutamine	Stop before conception or immediately on pregnancy confirmation

Consulting other medical professionals: Most maternal and fetal death occurs due to adverse effect of SCD rather than any obstetrical causes in pregnant women with SCD. So, a complete evaluation must be done evaluating respiratory, cardiovascular, and renal systems [22].

**Figure 3:**
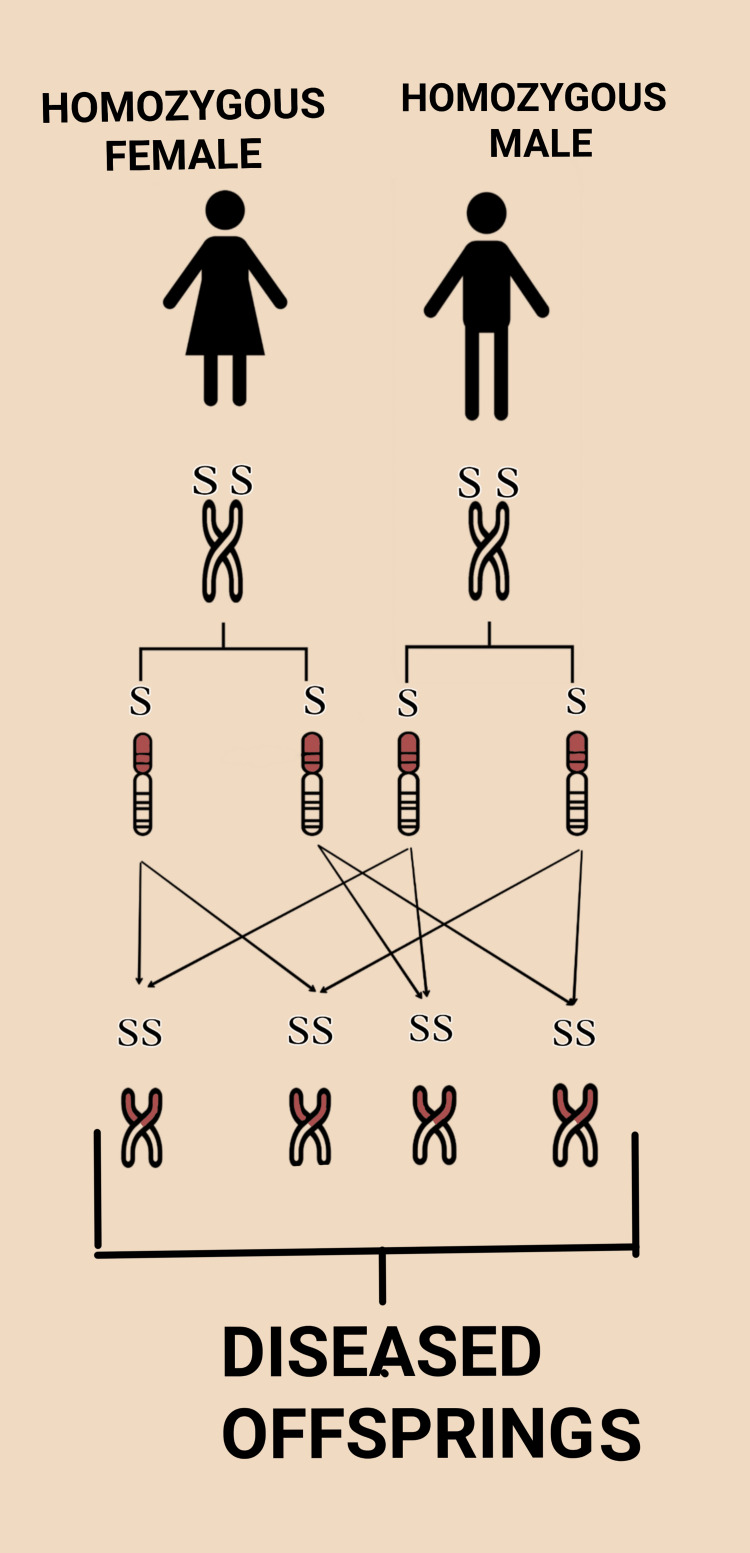
Risk of SCD offspring is 100% when both parents have SCD SCD: sickle cell disease Figure created by authors

**Figure 4:**
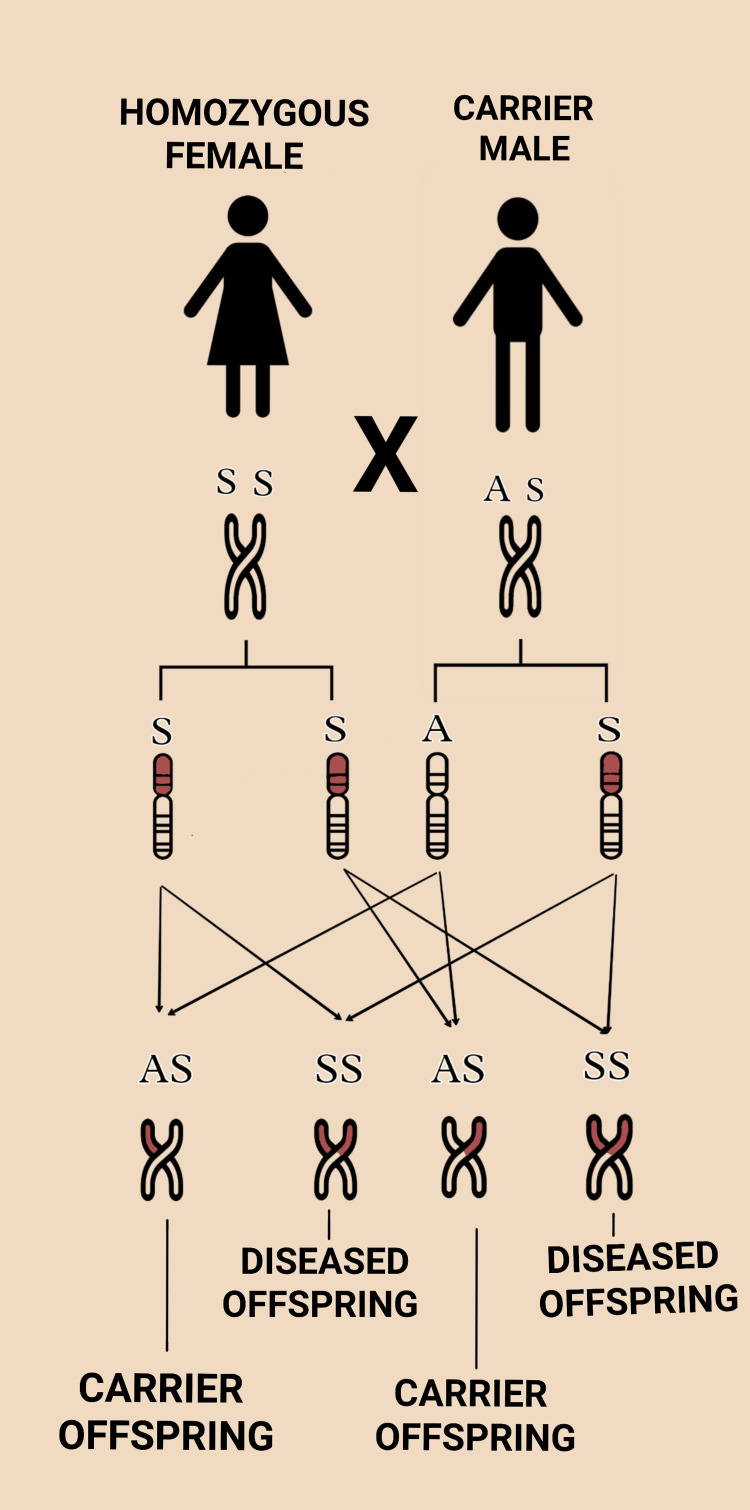
Fifty percent chances of diseased (SCD) offspring when one of either parent is the carrier and the other is homozygous Figure created by authors

Information: Couples and their relatives should be made aware of SCD and its adverse outcome associated with pregnancy. A patient must be evaluated for psychosocial aspects and socioeconomic status and should be informed about various family planning methods to reduce obstetric risk [27]. Even though there is extreme variation in the phenotype of SCD, particular laboratory investigations like high white blood cell count, high platelet count, and low oxygen saturation, as well as some clinical findings and history of twin pregnancy, age greater than 25 years, menarche after 14 years of age, repeatedly painful or sickle crisis help in the prediction of the course of outcome of pregnancy [34]. Couples should be made aware that pregnancy in SCD increases the risk to both maternal and fetal life. Premarital screening, preconceptual counseling, antenatal screening, good care during the antenatal period, and neonatal screening are all essential to reduce adverse outcomes [35]. For women with SCD planning to conceive, it is essential to make aware of the significance of partner screening by hemoglobin electrophoresis study. Also, the probability of the offspring’s risk of carrying a mutated gene for SCD should be assessed. The risk of passing on to offspring with sickle hemoglobinopathy is summarized in Figure 3 and Figure 4 [32].

All patients planning to conceive should pay attention to general health status for the best pregnancy outcome. The following is a comprehensive list of recommendations: yearly administration of influenza vaccine and a booster dose of streptococcus vaccine every five years; screening for asthma; screening for renal and liver function using kidney function test (KFT) and liver function test (LFT), respectively [32].

Antenatal Care

At the first antenatal visit, women must be evaluated using routine blood investigations and urine analysis during pregnancy. Mothers should be made aware of frequent and regular antenatal visits. The patient is advised to visit every other week for a routine checkup for the first and second trimesters. Mothers are advised to avoid precipitating factors resulting in sickle cell crisis as much as possible such as exposure to high temperature, low fluid intake, and excessive work. Also, frequent vomiting can lead to dehydration and may precipitate a sickle cell crisis. So, she must get treated for that and follow the physician's advice [19].

At each visit, the woman is assessed for blood pressure, and urine analysis should be done to rule out eclampsia, pre-eclampsia, and urinary tract infection (UTI), as patients with SCD are more prone to these [36]. Women at high risk for pre-eclampsia should take low-dose aspirin 75 mg from 12 weeks of pregnancy unless they are sensitive to aspirin [37]. Patients at antenatal visits in the first and second trimesters should be monitored for placenta previa, placental abruption, and preterm labor will help in the early detection of these complications, and it will be possible to manage them effectively. The patient should be prescribed multivitamins and folate (5 mg) supplements without iron in frequently transfused patients.

Between 24 and 28 weeks, monthly ultrasound should be done to assess fetal growth, and the patient asked to monitor for daily fetal moments count. From 32 weeks to 34 weeks, non-stress testing, as well as biophysical profile testing, should be done every week and between 28 and 30 for assessing umbilical artery flow and the ratio between systolic and diastolic for prediction of intrauterine growth retardation [33,38,39].

Some studies suggest that prophylactic transfusion in pregnant women reduces pain crisis and ACS but does not show much reduction in maternal or fetal outcomes despite the aggressive use of transfusion therapy. So, it is mainly recommended for increased SCD with severe complications, including ACS and severe acute anemia (hemoglobin less than 5 gm/dl). Preconceptual and antenatal recommendations are summarized in Table 4 [19,36,40].

**Table 4:** Recommendation in SCD pregnant woman SCD: sickle cell disease Table created by authors

	In normal pregnant women (non-SCD)	In SCD pregnant women
Folic acid	0.4 mg	5 mg
Vitamin D	May or may not be given	Must be given
Iron	60 mg of elemental iron every day	Prescribed if there is iron deficiency, otherwise not
Penicillin prophylaxis	Not needed	Recommended
Influenza vaccine	Not needed	Recommended
Pneumococcal vaccination	Not needed	Recommended

Intranatal Care

Delivery of females with SCD should be done in a hospital equipped with all facilities for efficiently managing high-risk pregnancies. Pregnancy should be continued till term with spontaneous labor onset without any obstetrical intervention unless there is an absolute indication for intervention. Normal vaginal delivery should be given preference over the cesarean section, which is reserved for obstetrical indication [40,41]. Adequate hydration and oxygen saturation should be maintained, and enough warmth should be provided to the patient [42]. Epidural analgesia is preferred over general anesthesia (GA) as GA increases the risk for ACS [26,40]. A patient may require more doses of analgesia than regular patients as there is increased tolerance to pain medication. Cardiotocography (CTG) is recommended for monitoring fetal distress, and cross-matched blood should be kept available at the delivery time [43].

Postnatal Care

During the postnatal period, assessment of loss of blood during labor and delivery is essential in patients with SCD, and transfusion therapy must be initiated if necessary. Fluid and electrolyte balance, along with oxygen saturation, should be maintained. In the event of a crisis, the management approach for patients should generally follow the established guidelines used for non-pregnant women. Non-steroidal anti-inflammatory drugs (NSAIDs) are safe and can be used for pain in the postnatal period. Exclusive breastfeeding should be encouraged [26]. Mothers who deliver by cesarean section have a higher incidence of surgical site infections, UTI, endometriosis, nausea, anorexia, and venous thromboembolism than normal vaginal delivery [20]. Twenty-four hours prior to delivery, prophylactic low molecular weight heparin (LMWH) should be discontinued and then initiated again after 12 hours of delivery. Hematological parameters, serum creatinine, transaminases, and bilirubin should be monitored daily [34,44].

Breastfeeding of neonate should be initiated as soon as possible after birth after evaluating the risk of delaying the re-introduction of any SCD drugs that crosses into milk. Drugs like hydroxyurea transfer through mother milk and are unsuitable for child health. So, it should be withheld during lactation. If there is a frequent vaso-occlusive crisis in the postpartum period or any history of frequent vaso-occlusive crisis, then hydroxyurea therapy would benefit. However, therapy is started only after careful discussion of the cessation of breastfeeding with the mother. Iron chelators should also be avoided [32,45,46].

Neonates should be screened for hemoglobinopathies and monitored for the adverse outcome of SCD. Progestogen-containing contraceptives and barrier methods are safe and effective in patients with SCD, and estrogen-containing pills can be used as second-line therapy [26].

Management of complications of SCD in pregnancy

*Painful* C*risis*

It is one of the common adverse effects, with an incidence of approximately 50% in patients with SCD. Antenatal women with a painful crisis or vaso-occlusive crisis should be immediately hospitalized. It is essential to maintain proper hydration and recommend sufficient bed rest. For pain relief, it is recommended to take paracetamol and other NSAIDs. If the above treatment fails to relieve pain, one can use narcotic analgesics such as morphine or diamorphine. Nevertheless, the use of meperidine should be avoided as it has high toxicity and risk of convulsions. The patient must be monitored for 30 minutes after the onset of the crisis for respiratory rate, pain, and sedation [20].

*Acute* C*hest* *Syndrome *(*ACS*)

Any pregnant woman with SCD complaining about cough and chest pain must be evaluated for ACS, which can be effectively managed by oxygen therapy, hydration, appropriate antibiotics, and analgesics, and if the condition demands one can go for a blood transfusion [20].

*Pulmonary* *Embolism*

It should be suspected in women complaining of chest pain and hypoxia with a normal chest X-ray. If it is suspected in pregnant women, then the patient must be immediately initiated on LMWH until other investigation reports come [20].

Stroke

If any pregnant woman with neurological impairment should be suspected of stroke, only thrombolysis is insufficient to manage sickle stroke. So, exchange transfusion is required [20].

*Hematological* *Complication*

Blood loss, bone marrow suppression due to viral infections like parvovirus infection, and nutritional deficiency causing anemia is the most common complication in pregnancy. Stroke, ACS, and acute multiorgan failure can be decreased with the help of prophylactic blood transfusion and indicated when the hemoglobin level is less than 7 gm/dl [20].

Infections

Infections like UTIs and respiratory tract infections causing fever and acidosis result in increased in sickling and further worsening anemia and can be managed by antibiotics and oxygen therapy [20].

## Conclusions

With advancements in medical sciences and the availability of better healthcare facilities, pregnancy-associated maternal and perinatal mortality and morbidity have decreased. The adverse outcome of pregnancy such as hypertensive disorder of pregnancy (pre-eclampsia and eclampsia), preterm labor, prematurity, intrauterine growth retardation, miscarriage, ACS, UTI, painful crisis, low birth weight, venous thromboembolism, etc., can be managed with proper preconceptual, antenatal, and perinatal care. Screening couples for sickle cell before conception and screening neonates as soon as possible after birth helps make an adequate management plan. A multidisciplinary approach, including a general physician, obstetrician, and hematologist, helps in early diagnosis, preventing complications, and effective management. In conclusion, SCD has a complex pathophysiology involving the deformation of RBCs, vaso-occlusion, and inflammatory processes. Pregnancy exacerbates the complications of SCD, leading to increased risks for both the mother and the fetus. Proper management and monitoring are crucial to minimize these risks and ensure the best possible outcomes for pregnant individuals with SCD.
